# COVID-19 in corrections: Quarantine of incarcerated people

**DOI:** 10.1371/journal.pone.0257842

**Published:** 2021-10-05

**Authors:** Morgan Maner, Katherine LeMasters, Jennifer Lao, Mariah Cowell, Kathryn Nowotny, David Cloud, Lauren Brinkley-Rubinstein

**Affiliations:** 1 Department of Social Medicine, Center for Health Equity Research, University of North Carolina at Chapel Hill, Chapel Hill, North Carolina, United States of America; 2 Department of Epidemiology, Gillings School of Global Public Health, University of North Carolina at Chapel Hill, Chapel Hill, North Carolina, United States of America; 3 College of Social Work, Utah Criminal Justice Center, University of North Carolina, Salt Lake City, Utah, United States of America; 4 Department of Sociology, University of Miami, Coral Gables, Florida, United States of America; 5 Department of Behavioral Sciences and Health Education, Emory University, Atlanta, Georgia, United States of America; Texas A&M University College Station, UNITED STATES

## Abstract

Carceral settings in the United States have been the source of many single site COVID-19 outbreaks. Quarantine is a strategy used to mitigate the spread of COVID-19 in correctional settings, and specific quarantine practices differ state to state. To better understand how states are using quarantine in prisons, we reviewed each state’s definition of quarantine and compared each state’s definition to the Centers for Disease Control’s (CDC) definition and recommendations for quarantine in jails and prisons. Most prison systems, 45 of 53, define quarantine, but definitions vary widely. No state published definitions of quarantine that align with all CDC recommendations, and only 9 states provide quarantine data. In these states, the highest recorded quarantine rate occurred in Ohio in May 2020 at 843 per 1,000. It is necessary for prison systems to standardize their definitions of quarantine and to utilize quarantine practices in accordance with CDC recommendations. In addition, data transparency is needed to better understand the use of quarantine and its effectiveness at mitigating COVID-19 outbreaks in carceral settings.

## Introduction

On July 1, 2021 there were 33,333,002 COVID-19 infections and over 600,000 deaths in the United States (US) [1]. The US bears a disproportionate share of the world’s COVID-19 burden. Simultaneously, the US houses a disproportionate share of the world’s incarcerated population [2]. These two public health emergencies are interconnected [3]. COVID-19 has entered our country’s prisons, jails, and the communities most impacted by mass incarceration at astounding rates [4, 5].

As of July 1st, 2021, the infection case rate in the US prison system was 165 per 1,000, which was approximately two times that of the general population. Additionally, 2,659 incarcerated people have died from COVID-19 and the age adjusted mortality rate in prisons was 3.5 times that of the general population [4]. These stark disparities demonstrate how severe the toll of COVID-19 has been for people who are incarcerated.

Carceral settings have utilized a number of mitigation strategies, including the widespread use of quarantine [6]. The Centers for Disease Control and Prevention (CDC) released guidance for the mitigation of COVID-19 spread in correctional settings on March 25th, 2020. This guidance defines quarantine as “the practice of separating individuals who have had close contact with someone with COVID-19 to determine whether they develop symptoms or test positive for the disease” [7]. In correctional settings, guidance specifies that individuals should be quarantined in a single cell with solid walls and a door that closes and should know the purpose and duration of quarantine. The CDC also recommends that all new intakes should be quarantined for 14 days upon arrival, although this was amended from a previous directive that advised a 10-day quarantine period [7]. This revision was made on January 1st, 2021.

## Methods

To develop a better understanding of widespread quarantine use in prison facilities across the country, we 1) reviewed each state’s definition of quarantine and 2) for the states that provide publicly available data on quarantine, calculated the number and rate of people who have experienced quarantine and explored the association between quarantine rates and COVID-19 testing and case rates. For the 9 states providing publicly available data on quarantine, we descriptively assessed trends in quarantine, COVID-19 testing, and COVID-19 cases. This study was performed as part of the COVID Prison Project (CPP) [4]. The CPP collects daily data on numbers of COVID-19 cases, testing, and deaths in correctional settings and regularly provides COVID-19 policy information from the 53 prison systems in the United States (i.e., each state’s Department of Corrections (DOC), the Federal Bureau of Prisons (BOP), Immigration and Customs Enforcement (ICE), and Puerto Rico).

### Definitions of quarantine

Data on states’ definitions of quarantine pertaining to COVID-19 come from DOC websites and news articles. DOC websites were initially searched for these definitions. If they were not published on the DOC website, an internet search for news articles relating to the system’s quarantine practices was performed. As this data was publicly available, no identifying information was collected. DOC definitions of quarantine were compared to seven CDC recommendations for quarantine practices as of January 15th, 2021 (Table 1).

**Table 1:** CDC recommendations for quarantine in correctional settings.

CDC Recommendation	Grading Scheme	Variable Name
“Ideally, each quarantined individual should be housed in a single cell with solid walls and a solid door that closes.”	DOCs meet recommendation if single- cell housing is used when feasible.	Single-Cell Quarantine
“Quarantine for COVID-19 should last for 14 days after the exposure has ended.”	DOCs meet recommendation if the quarantine period lasts 14 days for intakes or the general population.	14-Day Quarantine
“Some facilities may also choose to implement a “routine intake quarantine,” in which individuals newly incarcerated/detained are housed separately or as a group for 14 days before being integrated into general housing. This type of quarantine is conducted to prevent introduction of SARS-CoV-2 from incoming individuals whose exposure status is unknown, rather than in response to a known exposure to someone infected with SARS-CoV-2.”	DOCs meet recommendation if intake quarantine is implemented for 14 days.	Intake Quarantine
“Quarantined individuals should be monitored for COVID-19 symptoms at least once per day including temperature checks.”	DOCs meet recommendation if individuals are monitored for symptoms at least once per day during quarantine at intake or in the general population.	Monitoring for COVID symptoms
“Quarantined individuals can be released from quarantine restrictions if they have not developed COVID-19 symptoms and have not tested positive for SARS-CoV-2 for 14 days since their last exposure to someone who tested positive.”	DOCs meet recommendation if it is clearly stated that quarantined individuals are released at the end of their 14-day quarantine if no COVID-19 symptoms or positive COVID tests are reported since their last exposure.	Release from quarantine unless contraindicated
“Keep a quarantined individual’s movement outside the quarantine space to an absolute minimum.”	DOCs meet recommendation if it is clearly stated that quarantined individuals will remain	Minimized movement outside of quarantine space
“If a quarantined individual leaves the quarantine space for any reason, they should wear a mask (unless contraindicated) as source control, if not already wearing one.”	DOCs meet recommendation if they are provided masks to wear outside of the quarantine space.	Provides mask to wear outside of quarantine space

DOCs were considered to follow these recommendations if they met the conditions listed in the “Grading Scheme” column.

### COVID-19 data

Since April 2020, the CPP has tracked and recorded data related to COVID-19 cases and testing at 53 prison systems. The CPP publishes a daily aggregate dataset examining COVID-19 in correctional facilities. Data are aggregated by CPP based on public reports by prison systems. Each day, counts are extracted from Department of Corrections websites and supplemented with information from media reports and press releases. In total, the CPP collects daily data for 53 prison systems (all 50 states, Puerto Rico, BOP and ICE. These data include COVID-19 case and testing data. These data also include the number of individuals in quarantine in systems that report this information. All quarantine data reported here matched the data gathered by the system with two exceptions. First, to calculate Oklahoma’s daily quarantine numbers, we did not include the number of asymptomatic inmates discharged prior to test results nor the number of positive inmates that were retested. Second, in Florida, the security and medical quarantine numbers provided by the state were summed. For prison population data, we used data collected by the Vera Institute of Justice [8]. Prison population counts were collected during the first quarter of 2020. When possible, we used the most recent count collected on April 30th/May 1st, 2020. Data from Washington is not available for this date, so we used a count from March 31st, 2020.

### Analysis

This is a mixed methods study utilizing content coding of policies and other public documents and descriptive quantitative analysis. The content analysis of policies and public documents involved comparing definitions of quarantine to published CDC recommendations for quarantine in correctional settings. Definitions of quarantine were analyzed for alignment with seven areas of CDC guidance for quarantine (Table 1). These areas of guidance included: single-cell quarantine, 14-Day quarantine, intake quarantine, monitoring quarantine individuals for COVID symptoms, releasing individuals from quarantine, minimizing movement outside of quarantine space, and provision of a mask to wear outside of the quarantine space. Full definitions of quarantine, source of definitions and date of publication from all 53 prison systems can be found in the S1 Table.

We descriptively assessed the number of individuals in quarantine for each DOC through January 15, 2021. All analyses were conducted at the state level. We calculated the maximum number of people in quarantine within each system as well as the crude daily quarantine rates for each of the 9 systems reporting quarantine data. Starting with the earliest date of quarantine data reporting for each DOC, we also calculated crude daily COVID-19 testing and case rates for each system. We then calculated a Spearman correlation coefficient, a non-parametric correlation coefficient, to descriptively assess the relationship between quarantine and both COVID-19 cases and testing.

## Results

**Fig 1:**
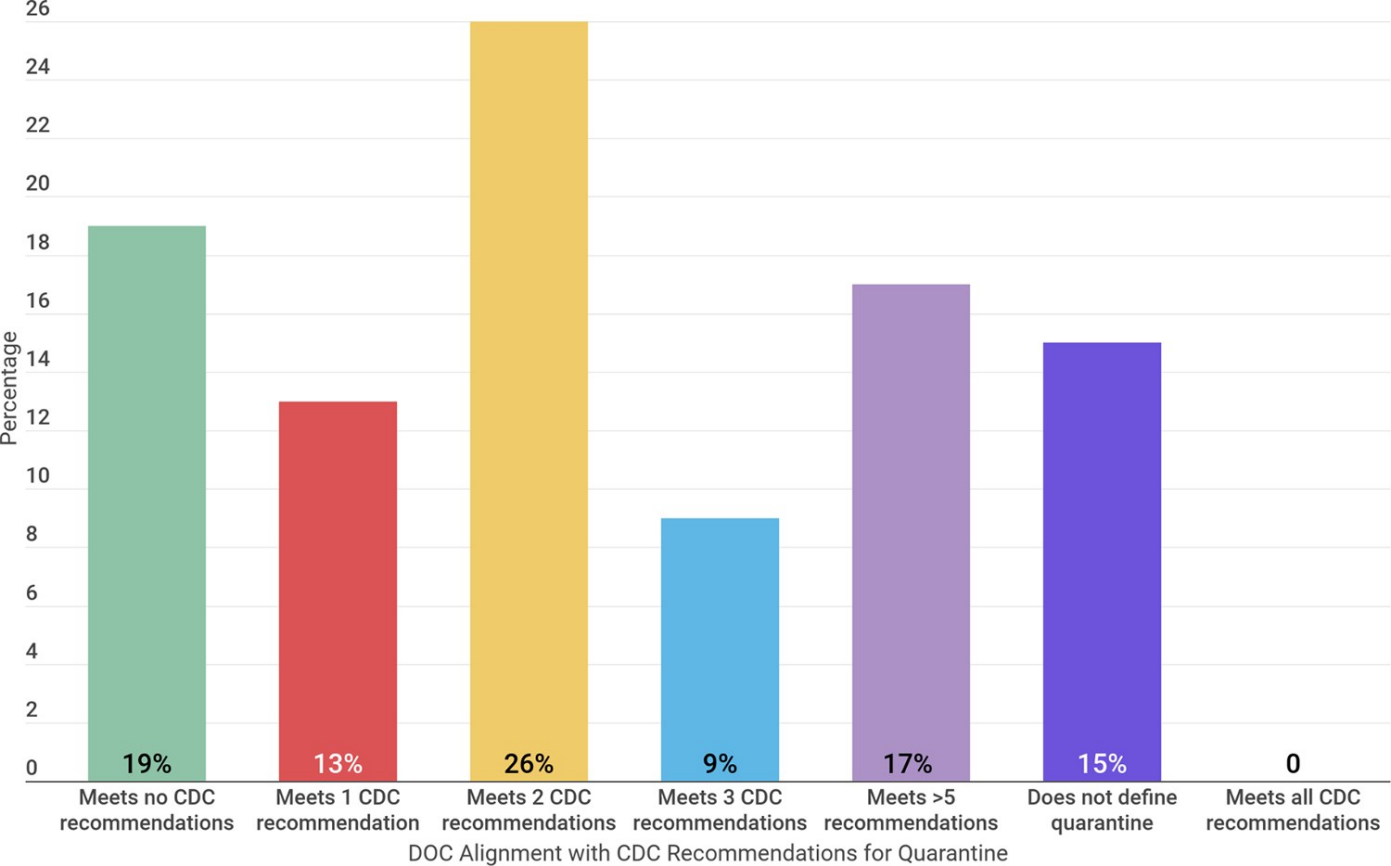
Percentage of DOCs that align with CDC recommendations according to DOC published definitions of quarantine.

Forty-five of the 53 prison systems provided a definition of quarantine; none of them follow all of the guidance the CDC has provided for these practices (Fig 1). Twenty-two systems presented definitions directly on their DOC websites, and 23 systems provided definitions in other locations (e.g., COVID-19 response plans, information sheets, frequently asked questions pages or through direct contact with the DOC). Two states, Oregon and Iowa, provided definitions in both formats. For example, Oregon defined quarantine alongside their COVID-19 data and provided more comprehensive information about their quarantine procedures through their “ODOC COVID-19 Infection Prevention, Testing, and De-Escalation Protocol” document [9].

Eight systems did not define quarantine on their website, in news articles or through direct contact with the research team at the time of analysis; these systems are ICE, Connecticut, New York, North Dakota, Tennessee, Virginia, Arizona, and Maine. Nevada DOC specifically notes that they are not following CDC guidance, stating that “these guidelines are agency specific and DO NOT mirror the current CDC guidelines. This is due to the fact that our inmates are a vulnerable population and we are a public safety agency with limited staff. Therefore, our margin of error is much less than the community’s” [10].

Although it is not specifically listed as a recommendation from the CDC, several systems noted the quarantine of high-risk or older individuals. These systems include Vermont, Louisiana, Pennsylvania and West Virginia. Vermont strictly mandates that “any inmate, aged 65 or older must be housed, and recreate, alone while on quarantine” [11]. Similarly, Louisiana DOC uses a “reverse isolation process” to quarantine high-risk and older incarcerated people; these individuals have separate housing and feeding areas from the general population [12]. Pennsylvania simply states, “All SCIs [state correctional institutions] identified vulnerable populations to keep them isolated” [13]. The criteria for who is considered a high-risk individual was not strictly defined by DOCs.

### Single-cell quarantine

**Fig 2:**
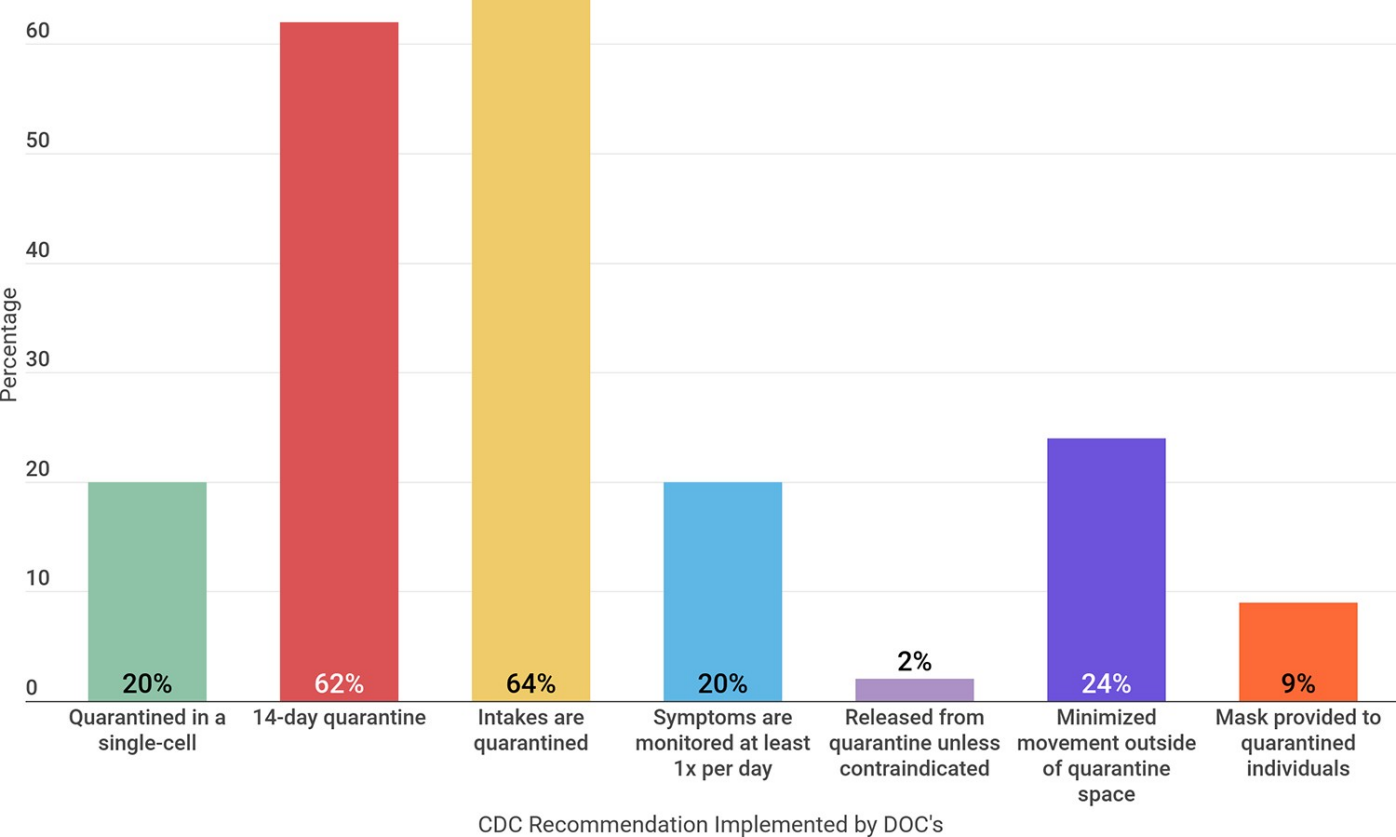
Percentage of DOCs that follow particular CDC recommendations according to published definitions of quarantine.

20% of systems reporting definitions of quarantine specified using single-cell quarantine when possible (Fig 2). These systems are Illinois, Indiana, BOP, Montana, Oregon, Vermont, West Virginia, Kansas and Hawaii. West Virginia notes that single-cell quarantine should be used for incarcerated people aged 60 or older; it is not clear that single-cell quarantine is used for the rest of the population [14]. Illinois used the term “administrative quarantine” to refer to “an intentional form of restricted movement within a facility to accommodate for unusual needs or circumstances, such as a pandemic outbreak” [15].

### Fourteen-day quarantine

Twenty-eight systems quarantine individuals for 14 days. Contrary to CDC guidance, California and Puerto Rico report quarantining individuals for 21 days, and Nevada reports quarantining individuals for 10 days.

### Intake quarantine

Twenty-nine systems specify conditions of mandatory quarantine for admissions or transfers (intake quarantine). For example, in Louisiana, each intake is “screened and assessed for symptoms, and then quarantined for 14 days before being placed in the general population.” Only individuals who must stay in state prison are accepted into the facility [12]. Contrary to CDC guidance, Vermont DOC quarantines intakes with the rest of the incarcerated population, stating “as the precautions for medical and intake quarantine are identical, the same location will be used” [11].

Until December 18th, 2020, Florida referred to quarantine of admissions or transfers as “security quarantine,” mandating that incarcerated people who are “asymptomatic and entering the facility from outside court, a community/work release center, county jail” needed to quarantine. Along with other significant changes in data reporting, quarantine is now simply defined by the Florida DOC as “[restricting] the movement of people who were exposed to a contagious disease or virus to see if they become sick” [16].

### Monitoring for COVID-19 symptoms

Nine systems report checking quarantined incarcerated individuals for COVID-19 symptoms at least once per day. These systems are: West Virginia, Mississippi, Alabama, Alaska, Colorado, BOP, Vermont, Kansas, and Hawaii. Alaska DOC noted the most symptom checks with three “rounds in all living areas, checking on the health status of well inmates” [17].

### Release from quarantine

Two systems, Hawaii and Oregon, specified that individuals can be released from quarantine at the end of the specified time period unless it is medically contraindicated.

### Minimized movement outside of quarantine space

Eleven systems indicate that movement outside of the quarantine space is kept to a minimum. These systems include: Hawaii, Colorado, Kansas, Montana, Alabama, Alaska, Nebraska, Indiana, New Hampshire, Illinois and Idaho. Montana indicates “all staff need to be diligent in monitoring that inmates in quarantine stay in the quarantined area and that inmates not in quarantine remain out of the area. In addition, it is important that all staff and inmates respect the health and safety of others. Quarantined inmates will be treated with respect and without harassment” [18].

### Mask provisions for quarantined individuals

Four systems indicate that masks are provided for incarcerated individuals: Hawaii, Colorado, Rhode Island and Montana. Colorado and Rhode Island DOC are specifically providing KN95 masks for incarcerated individuals [19, 20]. Montana explicitly mandates that incarcerated individuals are required to wear a mask any time they leave a quarantined space [18].

### Volume of people in quarantine

As of January 15, 2021, 9 systems had provided information on COVID-related quarantine of incarcerated people. These state systems are Florida, Indiana, Hawaii, Ohio, Oklahoma, Oregon, Washington, West Virginia, and Wisconsin. States began reporting quarantine numbers at different dates. While most began reporting in April 2020, Wisconsin did not begin reporting until July of 2020, and all states reported quarantine data through January 15, 2021 (Table 2).

**Table 2:** First reported date of quarantine data by system.

System	First Reported
Florida	April 22, 2020
Indiana	May 6, 2020
Hawaii	April 14, 2020
Ohio	March 26, 2020
Oklahoma	April 27, 2020
Oregon	May 7, 2020

In all 9 states, the date that states reached their maximum number and rate of quarantine varies (Table 3). For example, Ohio reached its maximum for quarantine in mid-May whereas Indiana, Washington, and Wisconsin had their maximum number of individuals in quarantine in December. The number of individuals that each state has quarantined at one time varies widely. The maximum number of individuals quarantined in a state on a given day ranges from 970 in Indiana (36.3 incarcerated persons per 1,000) to 40,827 in Ohio (843 incarcerated persons per 1,000). Ohio has the highest maximum quarantine rate at 843 per 1,000.

**Table 3:** Maximum numbers and rates for quarantine data.

System	Maximum Date	Maximum Number	Rate (per 1,000) at Maximum
Florida	08/11/2020-8/12/2020	23,386	253.0
Hawaii	10/31/2020-11/02/2020	1,041	244.0
Indiana	12/04/2020-12/06/2020	970	36.3
Ohio	05/19/2020-05/20/2020	40,827	843.0
Oklahoma	11/06/2020-11/08/2020	4,398	176.0
Oregon	09/29/2020	10,230	713.0
Washington	12/17/2020	3,866	206.0
West Virginia	10/14/2020	1,151	176.0
Wisconsin	12/17/2020	5,907	206.0

### The relationship between quarantine, COVID-19 testing, and COVID-19 cases

There is no correlation between quarantine and COVID-19 testing (R = 0.016; p-value: 0.80) or COVID-19 cases (R = -0.03; p-value: 0.62) across states. That is, quarantine rates do not consistently track with COVID-19 testing or COVID-19 case rates across states. In some states, there are clear patterns with quarantine being used in response to a spike in COVID-19 testing or cases. In other instances, patterns of quarantine result in a decrease in COVID-19 testing or cases. However, these trends are inconsistent and are varied.

## Discussion

COVID-19 outbreaks have devastated prisons across the country. The use of quarantine in correctional institutions has been largely reported as a COVID-19 mitigation strategy, but how these conditions have been used, how they are defined, and the number of people in quarantine at any given time remains largely unclear. According to published definitions of quarantine, it is unclear whether all DOCs are following CDC recommendations regarding these practices. Additionally, the sources of these definitions are disparate for each system.

Quarantine trends do not mirror those of COVID-19 testing and cases across states, making it unclear whether quarantine policies and practices are resulting in fewer COVID-19 cases and deaths. Quarantine is often used in response to high case counts, and thus COVID-19 transmission is already widespread. Additionally, given the lack of standardization in both definitions of and use of quarantine during the COVID-19 pandemic, interpreting trends in quarantine and its relationship to COVID-19 testing and cases is not feasible. It is important for states to better define these COVID-19 mitigation measures and for all states to report their definition and use of quarantine during the COVID-19 pandemic. Additionally, states have not consistently published information on quarantine policies by facility. This makes the information about facility trends opaque, as we are unable to track how quarantine practices map onto policies.

Clear explanations of how and why particular quarantine practices are used should be apparent to the public, specifically to friends and family of incarcerated people. The majority of family members with an incarcerated loved one report that their loved one lacks adequate protective equipment to prevent the spread of COVID-19 and access to medical care [21]. Family members have also reported increased levels of stress, anxiety, and worry of losing their loved one to COVID-19 while they are incarcerated [21]. It is thus critical for systems to be transparent with the strategies they are using to decrease transmission of COVID-19.

While containing the spread of COVID-19 requires quarantine in carceral settings, these conditions often mirror those of solitary confinement. While prisons and jails have been instructed to not use the same physical spaces for COVID-19 containment as they do for disciplinary purposes, this is often not the case. Additionally, depending on the DOCs definition, quarantined individuals may be unable to communicate with loved ones. There has long been concern over the use of solitary confinement in carceral settings [22]. The United Nations Standard Minimum Rules for the Treatment of Prisoners (“the Nelson Mandela Rules”) defines solitary confinement as the confinement of prisoners for 22 hours or more a day without meaningful human contact, with prolonged solitary confinement being 15 days or longer [23]. It has been well-documented that solitary confinement is over-utilized as a punitive measure in U.S. prisons [22, 24]. There are also negative, long-lasting mental and physical consequences of solitary confinement including high risk of self-harm during incarceration and overdose within two-weeks post-release [25–28].

Thus, while quarantine may increase public safety by limiting the spread of COVID-19, this practice can severely harm an individual’s health. Scholars and advocates have warned that the increased use of solitary confinement for COVID-19 quarantine jeopardizes the progress that has been made in recent years in ending the use of solitary confinement as a form of punishment [24]. Additionally, as incarcerated individuals are likely to be placed in solitary confinement for COVID-19 quarantine, they are disincentivized from reporting COVID-19 symptoms, potentially further contributing to COVID-19 spread and defeating the purpose of containing COVID-19 through quarantine. The ethical use of quarantine in carceral settings for COVID-19 containment requires that individuals have access to resources (e.g., tablets to communicate with loved ones) and medical staff at a minimum. It also necessitates frequent COVID-19 testing, adherence to other COVID-19 protocols (e.g., masking), the prioritization of vaccinations in carceral settings, and decarceration so that strict solitary measures are no longer in such high demand [28].

### Limitations

Given DOCs variable definitions of quarantine and policies on testing during the COVID-19 pandemic, it was not feasible to conduct formal statistical analyses to test associations between quarantine and COVID-19 testing and cases. If definitions, data reporting, and testing policies are standardized across systems, future analyses will be done to better understand the relationship between quarantine in carceral settings and COVID-19. Furthermore, COVID-19 outbreaks may be a more relevant indicator for quarantine use than testing and cases alone. However, consistent definitions of outbreaks in prisons and sufficient data on prisons’ capacities and living environments (e.g., dormitory, single cell) that would contribute to outbreaks are unavailable.

DOC definitions were compared to CDC guidance as updated on January 1st, 2021. A more recent update to CDC guidance on March 18th, 2021, provided clarification for quarantine duration for vaccinated staff and incarcerated individuals. At the time of this analysis, vaccinations were not implemented at all DOCs, and thus it would have been inappropriate to use this updated guidance for comparison. While the rapidly evolving nature of COVID-19 policies is important, this historical analysis provides insight into how DOCs did or did not share mitigation strategies, such as quarantine, with the public during the critical first year of the COVID-19 pandemic.

Federal, county-level and facility-specific guidelines were not analyzed, and quarantine practices are generalized as each state’s DOC reports the practice. Minnesota DOC was the only system that reported facility-specific guidelines. It is certainly possible that more systems realistically practice quarantine differently at each facility, however the fact that this information is not publicly available only speaks to the lack of data sharing and need for accountability.

Although this report defines the policies made publicly available on quarantine policies, it is not able to speak to the implementation of these policies. Policies cannot and are not always implemented to the full extent of their original intent. For example, at the end of 2018, the prison custody population exceeded their number of beds in over 25 states, removing social distancing as an option [29]. This paper focused on quarantine alone rather than medical isolation, defined as separating someone from the general population due to confirmed or suspected COVID-19 infection, because of the large variety of definitions used and the much more widespread use of quarantine [7]. Medical isolation during COVID-19 is critically important, particularly as it closely mirrors conditions of solitary confinement [22]. Future work should expand on understanding this practice and its relationship to COVID-19 spread.

## Conclusion

There are wide disparities in prison systems’ definitions of quarantine and in their data transparency. Correctional facilities must clearly communicate the strategies and practices they are implementing to mitigate COVID-19, particularly when they are not aligned with CDC recommendations. Rates of quarantine vary widely among the 9 states reporting these data and no trends in quarantine and COVID-19 testing and cases were identified across states. Both standardized definitions of quarantine and transparent data reporting across systems are critical for understanding quarantine practices, understanding their relationship to COVID-19 transmission, and holding carceral systems accountable for the health and safety of those incarcerated.

## Supporting information

S1 Table DOC definitions of quarantine. (DOCX)

S1 File References: DOC definitions of quarantine. (DOCX)
