# Supplementary material for: COVID-19 in corrections: Quarantine of incarcerated people
Source: PLoS One. 2021 Oct 5;16(10):e0257842. doi: 10.1371/journal.pone.0257842 (PMC8491943; doi:10.1371/journal.pone.0257842)
Supplement: S1 Table — (DOCX) [file pone.0257842.s001.docx]

| **System** | **Definition of Quarantine** | **Definition Source** | **Date last updated** |
| --- | --- | --- | --- |
| Federal Bureau of Prisons | “In the context of COVID-19, refers to separating (in an individual room or cohorting in a unit) asymptomatic persons who may have been exposed to the virus to (1) observe them for symptoms and signs of the illness during the incubation period, and (2) keep them apart from other incarcerated individuals.”  “The BOP utilizes three categories of quarantine: exposure, intake, and release/transfer”  “All BOP COVID-19 categories utilize a test-in/test-out strategy, with a quarantine duration of at least 14 days (the incubation period of the SARS- CoV2 virus).”  “To reduce the risk of transmission while in quarantine, facilities should make every effort to quarantine inmates individually in cells with solid walls and doors. Cohorting should only be practiced if there are no viable options to house them individually.”  “Inmates in exposure quarantine should be screened at least once daily for COVID-19 symptoms, including a temperature reading. Twice-daily screening is preferred when feasible.” | “Module 4. Medical Isolation and Quarantine” | 9/8/20 |
| U.S. Immigration and Customs Enforcement | None provided | ICE website | 3/15/20 |
| Alabama | “Inmates on level-two quarantine are restricted to their designated housing area for all activities including meals, and additional precautions and measures will be taken in accordance with CDC guidelines for COVID-19. Inmates will not be permitted to leave their designated housing area while under level-two quarantine unless movement absolutely is necessary and appropriate personal protective equipment is utilized.”  “Quarantined inmates will be monitored closely for signs and symptoms of COVID-19 to include taking and recording temperatures twice per day. Appropriate measures will be taken should an inmate become symptomatic in accordance with CDC guidelines for COVID-19.”  “Also last week, following the successful launch of the ADOC’s intake pilot program and the first cohort’s safe and secure transfer out of quarantine, a second cohort of 21 female inmates and 100 male inmates from county jails were received at the Tutwiler Quarantine Intake Facility and the Draper Quarantine Intake Facility, respectively. Per standing protocol, these new intakes will remain quarantined for a mandatory 14-day period before being transferred to their receiving correctional facilities” | AL DOC website - these are in ad-hoc updates; no formal definition provided. | 5/6/20 |
| Alaska | Quarantine: “the procedure of separating and restricting the movement of persons who are not sick yet but who were exposed. This allows rapid identification of those who will become sick.”  “The Alaska Department of Corrections may implement four levels of quarantine: 1) Individual; 2) Module; 3) Facility; or  4) Inter-Facility.” and “The institutional regional medical officer or health practitioner will authorize the appropriate level of quarantine.”  “All inmates in an affected area will remain on quarantine status for a minimum of 14 days, and inmates movement during this period will be minimized.”  “An inmate may be assigned to quarantine by a medical provider under authority granted by policy 804.01.” This policy is about administrative segregation and states, “Each institution shall have an administrative segregation unit to securely house inmates who require special supervision. The Department shall not use administrative segregation as punishment.”  “Rounds in all living areas, checking on the health status of well inmates in a quarantine area will be performed by medical or security staff at least three times a day.”  “All asymptomatic inmates will remain on intake quarantine (separate from the general population) until cleared for general population. In general clearance will require a negative COVID test and completion of a 14 days intake quarantine period.” | “Alaska DOC HARS COVID-19 Outbreak Response Plan” | 12/18/20 |
| Arizona | None provided | “Media Advisory: COVID‐19 Management Strategy Update” | 3/18/20 |
| Arkansas | “Facilities should utilize pre-designated isolation or quarantine areas for affected offenders to be  Housed.”  No further definition provided. | “COVID-19: Guidance for State Correctional Facilities and Local Detention Facilities” | 3/27/20 |
| California | “Quarantine all new arrivals for 14 days in cell based housing. Facilities which by design have no cell based housing shall house newly arriving inmates in cohorts of no more than 10 in  a dorm or small tent solely dedicated to the cohorts who arrive on the same day.”  “Inmate to remain in quarantine for at least 21 days, unless placement in quarantine is impossible (e.g.:  MSF), in which case the inmate will not be moved. Disposition to be determined in consultation with CME and public health.” | COVID-19 Screening and Testing Matrix for Patient Movement | 8/19/20 |
| Colorado | “Outdoor recreation, phone and video visitation will be made available in quarantined units to the extent possible once medical experts believe it is safe to do so, and depending on the extent of the outbreak in the individual facilities and on staffing allowances. The inmates will have access to their normal personal property during the quarantine period and will be able to send and receive mail.”  “If non-symptomatic, they are checked at least daily while on the active list. All symptoms are checked during this phase.  After 14 days, additional monitoring may be required, and the medical team will make that determination. Inmates have been quarantined based on whether or not they have tested positive, and/or have potentially been exposed. The inmates may remain in that quarantined status while the appropriate testing protocols are conducted. This helps to reduce the potential spread of the virus.”  “CDOC is providing KN95 masks to all staff members. Inmates are  also being provided masks, and are required to wear the masks. The CDOC is laundering the inmate masks regularly and has a stock of masks available to replace them when they wear out. Inmates can speak with a staff member about getting a replacement mask if it is needed.  Additional personal protective equipment is provided to both staff and inmates as needed.”  “Inmates will continue to remain with their cohorted groups until medical personnel feels that it is safe to adjust those cohort groups, and at that time the inmates may be moved back to their previous housing location, although that may not always be possible. The amount of time inmates are able to spend in outdoor recreation or in their pod area will vary depending on the facility, the unit that the inmate is housed in, the current part of the testing protocol they are in, clinical health concerns, and other factors.  Allowing inmates pod time and outdoor recreation is important to the Department, but it must be done in a way that still protects health and safety.” | “12-16-2020 FAQs on CDOC and COVID-19 Prevention and  Response” | 11/06/20 |
| Connecticut | None provided | “COVID Info Sheet” for incarcerated people | Unknown |
| Delaware | “Every correctional facility established quarantine tiers in preparation for COVID.” | “How the Delaware Department of Correction is containing COVID-19” | Updated 6/3/20 |
| Florida | “Quarantine separates and restricts the movement of people who were exposed to a contagious disease or virus to see if they become sick.” | FL DOC website | Unknown |
| Georgia | “Non-symptomatic offenders will be placed in 14-day security quarantine while awaiting test results. Those offenders receiving negative test results will resume the normal diagnostic process.”  “All new arrivals (intakes) and court return offenders will be administered a COVID-19 test. Offenders who are symptomatic will be placed in a designated medical isolation unit while awaiting test results. Non-symptomatic offenders will be placed in 14-day security quarantine while awaiting test results. Those offenders receiving negative test results will resume the normal diagnostic process.” | GA DOC Website | Updated 1/28/21 |
| Hawaii | “Quarantine: refers to the procedure of separating people who might have been exposed to COVID-19 from others.”  “Inmates who are close contacts of a suspected or confirmed COVID-19 case (i.e., other inmates,  staff, visitors, vendors, volunteers), should be placed under quarantine for 14 days...the duration of quarantine for COVID-19 is the 14-day incubation period. If a new case requiring medical  isolation is identified in the quarantine unit, then the 14-day quarantine period starts again.”  “Facilities should make every effort to quarantine close contacts of COVID-19 individually. Cohorting  multiple close contacts in quarantine could result in the transmission of COVID-19 to inmates who  are not infected. Cohorting should only be practiced if there are no other available options.”  “Restrict quarantined inmates from leaving the facility (including transfers to other facilities) during the 14-day quarantine period, unless released from custody or a transfer is necessary for medical care, infection control, lack of quarantine space, or extenuating security concerns.”  “CDC recommends monitoring inmates in quarantine at least once per day for COVID-19 symptoms and temperature. If an inmate develops symptoms for SARS-CoV-2, the inmate should be considered a suspected COVID-19 case, given a mask...and moved to medical isolation immediately…”  “Implement Routine Intake Quarantine of new admissions to the facility for 14 days before housed with the existing population, if possible.” | “HI DPS Pandemic Response Plan” | Updated 12/11/20 |
| Idaho | “If a COVID-19 test is positive, the area will remain quarantined for 14 days and all people living/working in that unit will be observed for the development of any symptoms. If any of the people in quarantine develop symptoms, the process begins  again, and the area stays in quarantine for another 14 days.”  Everyone is medically-screened before the transport and then all new individuals are going to one of two facilities (one for men, one for women) for the intake process. At the start of the crisis, IDOC instituted a 72-hour quarantine for all newly-admitted individuals. On April 20th, however, we initiated a full 14-day quarantine for all new admissions to IDOC. This process has reduced the number of individuals who can enter the IDOC system but greatly reduces the risk that someone new will introduce COVID-19 into our facilities. | “COVID-19 FAQ” | Updated 4/9/20 |
| Illinois | “Administrative Quarantine: All correctional facilities, Impact Incarceration Programs, and work camps are currently under Administrative Quarantine with no visits. Regular phone and video visit privileges will continue as normal. Administrative Quarantine is an intentional form of restricted movement within a facility to accommodate for unusual needs or circumstances, such as a pandemic outbreak.”  “If the COVID-19 test is negative, the transfer will proceed as scheduled. Upon arrival at the facility, the offender will be quarantined for 14 days.”  “All offenders shall be quarantined at the receiving facility in the pre-designated area for 14 days  prior to being assigned to a housing unit. Subject to availability, each offender in the predesignated intake quarantine area should be housed as follows: preferably, in a single cell with  solid walls and a solid door that closes; or, if that is unavailable, then in a single cell with solid 3 walls but without solid doors…” | IL DOC Website and letter from DOC director | Unknown |
| Indiana | Level 1: “Quarantine of an exposed individual to include single cell housing (bed, bath, solid door), in cell meals, restriction of movement, and separation from congregate activities for duration of incubation period (14 days). If mild symptoms self-monitor.”  Extra levels - system wide  “During a disease outbreak, all offenders shall be asked about symptoms at intake  and upon transfer. Offenders who report any symptoms of concern shall be separated from the general population to the extent possible until evaluated by  Health Services staff. The receipt of offenders from county jails with known outbreaks may need to be temporarily halted. This decision will be made by the Commissioner.” | “Indiana Department of Correction  Preparedness and Response Plan (Adult and Juvenile)” | 3/30/20 |
| Iowa | “Separates and restricts the movement of people who were exposed to a contagious disease to see if they become sick”  “All new intakes are placed in quarantined units and monitored for COVID-19 symptoms for 14 days prior to their movement” | “Fiscal Update Article: Department of Corrections — COVID-19 Response: March 2020 | DOC Website: 4/10/20 |
| Kansas | “If epidemiologic risk only (no symptoms), place inmate in single cell with BID monitoring for symptoms x 14 days (see  quarantine information on pages 2-4) and schedule for medical provider review.” Tiers quarantine based on level of risk. (Individual, Module, Facility and Inter-Facility) | “Prevention and Control of COVID-19 in Correctional and  Detention Facilities” | Published 4/30; Updated 11/19/20 |
| Kentucky | “If any new inmates are received at an institution, they are quarantined for 14 days prior to being incorporated with the general inmate population; anyone who has had close contact with that individual will be quarantined from the rest of the population.” | KY DOC Website - FAQs section | Unknown |
| Louisiana | “Limited....new intakes to only those who must be housed in state prison and facility to facility transfers. (Each intake is screened and  assessed for symptoms, and then quarantined for 14 days before being placed in general population).”  “Commenced Reverse isolation process of older and high-risk inmates. Designated a separate housing/ feeding location for reverse isolation. These inmates are also allowed into a recreation area reserved just for them to minimize their interaction with the rest of the facility.” | “LA. DPS&C  Summary of COVID-19  Response” | 12/2/20 |
| Maine* | None provided. |  |  |
| Maryland | “Close contact inmates without symptoms will be housed in designated quarantine area for 14 days and assessed by Inmate medical staff for new onset of symptoms.”  “All orders above shall have nursing parameters for monitoring of temperatures and symptoms two times a day for inmates identified to be quarantined.” | “Department of Public Safety and Correctional Services: OOS Information Bulletin | 5/20/20 |
| Massachusetts | “Masks are mandatory for all staff working in Department facilities and any inmate who enters a facility is quarantined for fourteen days.” | “The DOC’s Preparation and Response to COVID-19” | 5/20 |
| Michigan | “Prisoners who have been identified as having close contact with another prisoner who tests positive, but have not tested positive for the virus themselves, will be isolated from the general population at their facility for the 14-day quarantine period.”  “Prisoners at the Reception Center are on “quarantine” status, meaning they cannot receive visits from friends or family. These prisoners can receive visits from qualified clergy, the Office of the Legislative Ombudsman, and attorneys on official business. Prisoners may use the phone to place collect calls to family and friends when out-of-cell and given permission. Prisoners at the Reception Center receive limited daily outdoor recreation time. Library books and law books are available, along with religious services.” | MI DOC Website | Unknown |
| Minnesota | Each facility has specific guidelines. MCF-Shakopee facility for example: “All women incarcerated in Minnesota come to MCF-Shakopee. All newly admitted women have their temperature taken, are screened for symptoms, and quarantined for 14 days upon arrival. New intake inmates are tested for COVID-19 on the 1st, 7th, and 12th day of quarantine prior to being moved to general population.” | MN DOC Website | Facilities have reviewed their guidelines at different dates. For example, MCF - Faribault last updated their plan 1/4/21 |
| Mississippi | “MDOC cohort quarantines all new admissions into the correctional facilities for 14 days to monitor for signs and symptoms of COVID-19. Additionally, following the return of hospital visits, incarcerated individuals are also quarantined for 14 days. Those with possible exposure to a COVID-positive individual discovered through contact tracing are also quarantined for 14 days and monitored for signs and symptoms of COVID-19.” | MDOC Website through (Q+A) PDF | Updated daily |
| Missouri | “In early March, the Department of Corrections began COVID-19 testing of offenders who exhibited symptoms of COVID-19 or may had contact with an infected person, following Centers for Disease Control & Prevention guidelines. Offenders are quarantined pending test results.” | MO DOC Website | Unknown |
| Montana | Individuals who have had close contact with a COVID-19 case will be assessed to determine whether they have developed symptoms of the disease. Those individuals will be quarantined up to 14 days, or as directed by medical staff. Monitoring will continue throughout the quarantine. Ideally, each individual would be quarantined in a single cell with solid walls and a solid door that closes. If sufficient cell space is not available, Health Services staff will determine which inmates may be housed together based on risk factors.”  “While under quarantine, inmates will always be  required to wear a face mask when they leave their rooms.”  “All staff need to be diligent in monitoring that inmates in quarantine stay in the quarantined area and that  inmates not in quarantine remain out of the area. In addition, it is important that all staff and inmates respect the health and safety of others. Quarantined inmates will be treated with respect and without harassment.”  “Staff will conduct routine screening of staff and residents to detect Covid-19 symptoms as soon as possible. Screening includes temperature checks and completion of appropriate screening tools/symptom checks.” | “Montana Department of Corrections: Plan for Preventing the Spread of Disease  in Secure and Other Facilities | 6/20/20 |
| Nebraska | “All new admissions (new commits, safe keepers, parole/PRS violators) to NDCS reception facilities are placed on quarantine status for a minimum of 14 days unless an exception is made by a facility health care manager.”  “Precautions will be undertaken to ensure the proper care of individuals who are sick, as well as to maintain the health and well-being of staff who are in contact with them. This includes requiring the use of PPE by staff members, utilization of masks, delivery of meals and medications to and from cells, exclusion from group activities and maintaining quarantine/isolation status until the person is cleared by a medical provider.” | NDCS Website - FAQ Section | Unknown |
| Nevada | “Offenders who have been housed outside of the NDOC for greater than 24 hours transferring into NDOC facilities will require a minimum 14-day quarantine and a COVID-19 swab test on day 7 after their transfer or sooner if they develop any symptoms. For the offender to return to the general population they will require a minimum 14-day quarantine with 72 hours free of symptoms with a negative COVID-19 test result.”  “Please note that these guidelines are agency specific and DO NOT mirror the current CDC guidelines. This is due to the fact that our inmates are a vulnerable population and we are a public safety agency with limited staff. Therefore our margin of error is much less than the community's. If an inmate tests positive for COVID-19 they will be quarantined (negative airflow room if symptomatic) for 10 days with at least 72 hours free of any symptoms. Prior to return to general population they will require TWO negative COVID-19 test results (at least 48 hours apart) and no symptoms.” | NDOC Website | Unknown |
| New Hampshire | “The NHDOC utilizes the recommended functions of quarantine and medical isolation as outlined in CDC guidance and has plans by facility based on physical plant.”  “Necessary transfers to NHDOC facilities are  quarantined and triaged by healthcare staff.” | NH DOC website - FAQ section | Unknown |
| New Jersey | “As per the Centers for Disease Control and Prevention (CDC) guidance, individuals who have contact with a person who has COVID-19 are required to be quarantined for 14 days. Contact is defined as someone who was within 6 feet of an infected person for a cumulative total of 15 minutes or more over a 24-hour period. The fifteen minutes of cumulative exposure at a distance of 6 feet or less (2 meters) triggers the Department’s contact tracing notification protocols.”  “Inmates with known exposure to COVID-19 shall be quarantined in a specifically designated quarantine unit within the facility. The inmates shall be monitored by medical staff. If the inmate becomes symptomatic during this 14-day quarantine period, they will be evaluated for medical isolation or referred to a hospital for evaluation and testing...even when an individual is in medical isolation or quarantine, access to communication devices are made available and sanitized after each use.”  “JPay kiosk usage and access to U.S. mail postage, free of charge, to maintain ties to loved ones during visitation suspension. The Department continues to offer these services. Even when an individual is in medical isolation or quarantine, access to communication devices are made available and sanitized after each use.” | NJDOC website | 10/20/20 |
| New Mexico | “All new intakes at our Central New Mexico Correctional Facility (male intake prison) and Western New Mexico Correctional Facility (female intake prison) are subject to immediate 14-day quarantine. Once they produce a negative test, they are then integrated to the intake process and further into the general population. Additionally, all interfacility transfers/movement (this applies to all of our 11 prison facilities) are quarantined for 72 hours upon arrival to their new facility, tested, and once a negative result is produced, they are put into (the) general population. If an inmate exhibits symptoms and is tested, or if there is known exposure to a positive individual, inmates are placed in quarantine units until they produce a negative test result.” | Per Public Information Officer at NMCD | 9/18/20 |
| New York | None provided. | NY DOC Website | Unknown |
| North Carolina | “A 14-day quarantine period for all incoming offenders from county jails to help prevent the introduction of COVID-19 to the prison system.” | NC DPS Website | Unknown |
| North Dakota | None provided | ND DOC website | Unknown |
| Ohio | “Quarantine separates and restricts the movement of people who were exposed, or potentially exposed,  to a contagious disease to see if they become sick.” | OH DRC Website | Updated Daily |
| Oklahoma | “Quarantine is similar to isolation except that it is used to separate people who are not yet ill (but who have been exposed) from others so that transmission is prevented.” | “Pandemic Planning Guide” | 3/16/20 |
| Oregon | Quarantine refers to confining individuals who have had close contact with a COVID-19 case to determine whether they develop symptoms of the disease. Quarantine for COVID-19 lasts for a period of 14 days. Ideally, each quarantined individual would be quarantined in a single cell with solid walls and a solid door that closes. If symptoms develop during the 14-day period, the AIC will be placed under medical isolation and evaluated for COVID-19. If symptoms do not develop, movement restrictions can be lifted, and the individual can return to their previous residency status within the facility.” A tiered quarantine protocol exists as well. It ranges from individual to system-wide quarantine. | ODOC Website and “ODOC COVID-19 Infection Prevention, Testing, and De-Escalation Protocol” | Tiered quarantine: updated 7/14/20 |
| Pennsylvania | PA employs a tiered quarantine that depends on the amount of cases in each facility (see Demobilization plan)  “To help mitigate the mental health aspect of COVID-19 quarantine, inmates have been given additional controlled out of cell time, video visitation, free phone calls, an increased amount of free mailing envelopes, COVID-19 specific information shared on inmate channel, inspirational videos from celebrities and free cable for those who have TVs. Some institutions have purchased board games and puzzles for the inmates as well and all institutions have increased/frequent rounding of the blocks by executive staff talking to the inmates to hear about their concerns.”  They also say “When quarantines happen, individuals housed in those housing units cannot participate in video visitation for the duration of the lockdown….”  “All SCIs [state correctional institutions] identified vulnerable population to keep them isolated.” | “Pennsylvania Department of Corrections  Demobilization Plan  COVID-19  Inmate Movement” | 5/20/20 |
| Puerto Rico | Suspected and confirmed cases will follow similar protocol with a 21 day quarantine period. | “Prevención y Manejo de Infecciones por Coronavirus COVID-19”  (Prevention and Management of COVID-19 Infections) | 3/20/20 |
| Rhode Island | “Quarantine is used to keep someone who might have been exposed to COVID-19 away from others. Quarantine helps prevent spread of disease that can occur before a person knows they are sick. People in quarantine should separate from others, their health is monitored regularly, and they should follow directions given by their healthcare provider. Quarantine Period is 14 days from commitment to the Adult Correctional Facilities (ACI), or from last date of contact with a COVID positive person.”  “Masks (KN95) must be worn by inmates on quarantine whenever out of their cell.” | “RHODE ISLAND DEPARTMENT OF CORRECTIONS  COVID-19  Inmate Quarantine & Isolation Protocols” | Updated 12/9/20 |
| South Carolina | “Admission of new inmates will continue; however, such  inmates will be screened, checked for exposure and isolated or quarantined as deemed appropriate.” | “South Carolina Department of Corrections (SCDC) COVID-19 Action Plan” | 3/16/20 |
| South Dakota | “Isolation and quarantine plans have been developed for all facilities.” No further detail reported.  “We are adjusting admission and intake processes to ensure individual assessment,  separation and cohorting of new admissions for their first 14 days in the facility.” | “COVID-19 Frequently Asked Questions” | 9/10/20 |
| Tennessee | None provided. | TN DOC Website | Unknown |
| Texas | Medical Restriction = “restriction of the movement of well offenders, who may have been exposed to COVID-19, for monitoring.”  Precautionary Lockdown = restriction of movement on a facility. Units with high rates of positive COVID-19 tests are placed on precautionary lockdown.” | Texas Department of Criminal Justice website | Unknown |
| Utah | Quarantine: Intakes will be in a cohort together for two weeks, and will each be tested for COVID-19 within 24-48 hours of arrival; positive cases can lead to a “potential” facility-wide lockdown | “COVID-19: What to expect at the Utah Department of Corrections” | 4/7/20 |
| Vermont | “The purpose of Medical Quarantine is to ensure that incarcerated individuals who are known to have been exposed to the virus are kept separate from other incarcerated individuals to assess whether they develop viral infection symptoms.  “Intake quarantine: The physical separation of the persons lodged from the community and current inmates returning from an ER transport. Any inmate, aged 65 or older must be housed, and recreate, alone while on quarantine.”  “To minimize the likelihood of disease transmission to fellow quarantined persons,those who are placed in quarantine should be required to wear microfiber masks while in quarantine. Masks should be replaced as needed, if they become soiled, or at least every 8 hours.”  “Three times daily, medical staff will assess whether inmates in quarantine should be screened for symptoms including subjective fever and a temperature. Symptomatic patients need to be isolated or cohorted.”  “As the precautions for medical and Intake Quarantine are identical, the same location will be used.” | “Vermont Department of Corrections COVID Guidelines - Facility” | Updated 12/30/20 |
| Virginia** | None provided |  |  |
| Washington | “Patients who are asymptomatic but have been in close contact with confirmed or suspected COVID-19 patients should  be placed on quarantine status...If patients are in medical isolation or quarantine, allowances will be made to accommodate patients: a) Television, playing cards and/or other recreational activities will be provided. b) There will be no cost to the patient for the duration of their stay. c) All patients/residents placed in medical isolation/quarantine will be issued hygiene kits and new clothing as needed.”  “Patients testing negative for COVID-19 will remain on quarantine status. They will be retested for COVID-19 on quarantine day #7. Patients testing negative for COVID-19 will remain on quarantine status until 14 days from the time of last contact with the index case has elapsed.”  “Within 24 hours of arrival patients in intake separation will be tested for COVID-19 If the COVID-19 test is negative and the patient is asymptomatic, the patient remains in intake  separation and is re-tested on day 7 after intake. If the second test is negative, the patient can be released to the general population on day 10 post intake.”  “Quarantined patients ideally should be housed alone or cohorted when determined by medical to be necessary with other quarantined patients from the same exposure.” | “WA State DOC COVID-19 Screening, Testing, and Infection Control Guideline  Version 23” | Updated 12/2/20 |
| West Virginia | “Confining asymptomatic persons who are contacts to COVID-19 while they are in incubation period (up to 14 days)”. If feasible, facilities should identify persons 60 and older or with comorbid conditions and, if possible, quarantine them in single cells.”  “At least daily, offenders in quarantine should be screened for symptoms including subjective fever, and a temperature. | “Policy Directive - COVID Response Plan | 3/20/20 |
|  | “Quarantine separates and restricts movement of people who were potentially or directly exposed to a contagious disease to see if they become sick.” | WI DOC Website | Unknown |
|  | “All new inmates coming into WDOC facilities are quarantined for 14 days upon arrival and  are tested twice during that period.” | “COVID-19 outbreak at Wyoming County prison could be harbinger of more to come”  Quarantine: “Wyoming Department of Corrections  COVID-19 Update” | 11/19/20 |

**S1 Table. DOC Definitions of Quarantine.** Note: *Maine does provide a link to a “Testing Plan document” on their DOC website, but it did not work at the time of this analysis. **VA did publish an operating procedure for infectious disease control in February 2019, but it had not been publicly referenced in relation to the COVID-19 pandemic at the time of this analysis.
