# Supplementary material for: COVID-19 in corrections: Quarantine of incarcerated people
Source: PLoS One. 2021 Oct 5;16(10):e0257842. doi: 10.1371/journal.pone.0257842 (PMC8491943; doi:10.1371/journal.pone.0257842)
Supplement: S1 File — (DOCX) [file pone.0257842.s002.docx]

Federal Bureau of Prisons. (2020). Module 4 Medical Isolation and Quarantine of COVID Pandemic Response Plan. [https://www.bop.gov/foia/docs//Mod_4_Inmate_Isolation_and_Quarantine_of_COVID_Pandemic_Response_Plan_08312020.pdf](https://www.bop.gov/foia/docs/Mod_4_Inmate_Isolation_and_Quarantine_of_COVID_Pandemic_Response_Plan_08312020.pdf). (Accessed 15 January 2021).

U.S. Immigration and Customs Enforcement. ICE Guidance on COVID-19. <https://www.ice.gov/coronavirus>.(Accessed 15 January 2021).

Alabama Dept of Corrections. COVID-19 Update. <http://www.doc.alabama.gov/COVID19NewsFeed>. (Accessed 15 January 2021).

State of Alaska Department of Corrections. (2020). Alaska DOC HARS COVID -19 Response Plan. <https://doc.alaska.gov/covid-19/docs/Alaska%20DOC%20HARS%20COVID-19%20Outbreak%20Response%20Plan.pdf?09022020>. (Accessed 15 January 2021).

State of Alaska Department of Corrections. (2020). Administrative Segregation. <https://doc.alaska.gov/pnp/pdf/804.01.pdf>. (Accessed 15 January 2021).

Arkansas Department of Health. (2020). COVID-19: Guidance for State Correctional Facilities and Local Detention Facilities. <https://doc.arkansas.gov/wp-content/uploads/2020/09/ADH_Guidance_-_COVID-19_Guidance_for_State_Correctional_Facilities_and_Local_Detention_Facilities_-_March_27_2020.pdf>. (Accessed 15 January 2021).

Arizona Department of Corrections, Rehabilitation and Reentry. (2020). COVID-19 Management Strategy Update. <https://corrections.az.gov/sites/default/files/notifications/adcrr_covid-19_management_strategy_update_3-18-2020.pdf>. (Accessed 15 January 2021). 

California Correctional Healthcare Services. (2020). <https://cchcs.ca.gov/wp-content/uploads/sites/60/COVID19/Appendix13-PatientMovement.pdf> (Accessed 15 January 2021)

Colorado Department of Corrections. COVID-19 FAQ and Updates. <https://www.colorado.gov/pacific/cdoc/covid-19-faq-and-updates>. (Accessed 15 January 2021).

Connecticut State Department of Correction. (2020). COVID-19 Information Flyer for Offenders. <https://portal.ct.gov/-/media/DOC/Pdf/Coronavirus-3-20/COVID-19-inmate-informational-sheet-033020.pdf>. (Accessed 15 January 2021).

Delaware Department of Correction. How the Delaware Department of Correction is containing COVID-19. <https://doc.delaware.gov/assets/documents/How_the_Delaware_DOC_is_containing_COVID19.pdf>. (Accessed 15 January 2021).

Florida Department of Corrections. COVID-19 Information. <http://www.dc.state.fl.us/comm/covid-19.html#confirmed>. (Accessed 15 January 2021).

The Georgia Department of Corrections. Frequently Asked Questions for Friends & Family. <http://www.dcor.state.ga.us/content/faq2>. (Accessed 15 January 2021).

State of Hawaii Department of Public Safety. (2020). Pandemic Response Plan COVID-19. <https://dps.hawaii.gov/wp-content/uploads/2020/09/PSD-COVID-19-Pandemic-Response-Plan-Revised-September-2020.pdf>. (Accessed 15 January 2021).

Idaho Department of Corrections. COVID-19 ALERT - Idaho Department of Correction. https://www.idoc.idaho.gov/content/document/covid_19_alert. (Accessed 15 January 2021).

Idaho Department of Correction. COVID-19 FAQs.   <https://www.idoc.idaho.gov/content/document/covid_19_faq>. (Accessed 11 January 2021).

Illinois Department of Corrections. COVID-19 Response: COVID-19 Intra-Agency Transfers and County Jail/Detention Center Intakes. <https://www2.illinois.gov/idoc/facilities/Documents/COVID-19/TransfersIntakes/COVID_19%20Intake%20and%20Transfer%20Procedures.pdf>. (Accessed 11 January 2021).

Illinois Department of Corrections. COVID-19 Response. <https://www2.illinois.gov/idoc/facilities/Pages/Covid19Response.aspx>. (Accessed 15 January 2021).

Indiana Department of Correction. IDOC Facility COVID-19 Statistics. <https://www.in.gov/idoc/about-idoc/idoc-facility-covid-19-statistics/>. (Accessed 15 January 2021).

Indiana Department of Correction. (2020). Preparedness and Response Plan (Adult and Juvenile). <https://www.in.gov/idoc/files/IDOC-Pandemic-Response-Plan-3-3-2020.pdf>. (Accessed 15 January 2021).

Iowa Department of Corrections. (2020). Department of Corrections - COVID- 19 Response: March 2020. <https://doc.iowa.gov/sites/default/files/documents/2020/03/lsa_note_on_doc_steps.pdf>. (Accessed 15 January 2021).

Iowa Department of Corrections. DOC COVID19 FAQ. <https://doc.iowa.gov/document/doc-covid19-faq>. (Accessed 15 January 2021).

State of Iowa Department of Corrections. (2018). Disease Specific Precautions.  <https://doc.iowa.gov/sites/default/files/hsp-905_disease_specific_precautions.pdf>. (Accessed 15 January 2021).

Kansas Department of Health and Environment. (2020). Prevention and Control of COVID-19 in Correctional and Detention Facilities. <https://www.coronavirus.kdheks.gov/DocumentCenter/View/346/Prevention-and-Control-of-COVID-19-in-Correctional-and-Detention-Facilities-PDF-9-10-20?bidId=>. (Accessed 15 January 2021).

Commonwealth of Kentucky Department of Corrections. FAQs. <https://corrections.ky.gov/Facilities/pages/covid19FAQ.aspx>. (Accessed 15 January 2021).

Louisiana Department of Public Safety and Corrections. Summary of COVID-19. <https://doc.louisiana.gov/wp-content/uploads/2020/04/DOC-Summary-of-COVID-19-Response-for-WEBSITE.pdf>. (Accessed 15 January 2021).

Maine Department of Corrections. (2020). DOC Facility Reopening Plan – August 2020. <https://www.maine.gov/corrections/sites/maine.gov.corrections/files/inline-files/COVID-19%20Facility%20Reopening%20Plans%20-%20August%202020_0.pdf>. (Accessed 15 January 2021).

Maryland Department of Public Safety and Correctional Services. Department of Public Safety and Correctional Services: OOS Information Bulletin. <https://itcd.dpscs.state.md.us/PIA/ShowFile.aspx?fileID=1520>. (Accessed 15 January 2021).

Massachusetts Department of Correction.  The DOC’s Preparation and Response to COVID-19. <https://www.mass.gov/doc/the-docs-preparation-and-response-to-covid-19/download>. (Accessed 15 January 2021).

Michigan Department of Corrections. MDOC Response and Information on coronavirus (COVID-19).<https://medium.com/@MichiganDOC/mdoc-takes-steps-to-prevent-spread-of-coronavirus-covid-19-250f43144337>. (Accessed 15 January 2021).

Minnesota Department of Corrections. Facility Specific COVID-19 Responses. <https://mn.gov/doc/about/covid-19-updates/facility-specific-covid-19-responses>. (Accessed 15 January 2021).

Mississippi Department of Correction. COVID-19 Questions and Answers. <https://www.mdoc.ms.gov/Documents/covid-19/QA-Questions%20and%20Answers.pdf>. (Accessed 15 January 2021).

 Missouri Department of Corrections. COVID-19 Update. <https://doc.mo.gov/media-center/newsroom/covid-19>. (Accessed 15 January 2021).

Montana Department of Corrections. (2020). Plan for Preventing the Spread of Disease in Secure and Other Facilities. <https://cor.mt.gov/Portals/104/COVID-19/doc%20facility%20plan6.19.20.pdf?ver=2020-06-19-113749-303>. (Accessed 15 January 2021).

Nebraska Department of Correctional Services. NDCS Frequently Asked Questions. <https://corrections.nebraska.gov/ndcs-frequently-asked-questions>. (Accessed 15 January 2021).

State of Nevada Department of Corrections. NDOC COVID-19 Updates. <http://doc.nv.gov/About/Press_Release/covid19_updates/>. (Accessed 15 January 2021).

New Hampshire Department of Corrections. FAQs. <https://www.covid19.nhdoc.nh.gov/faqs>. (Accessed 15 January 2021).

New Jersey Department of Corrections. COVID 19 Updates. <https://www.state.nj.us/corrections/pages/COVID19Updates.shtml>. (Accessed 15 January 2021).

New Mexico Corrections Department. COVID-19 Updates. <https://cd.nm.gov/covid-19-updates/>. (Accessed 15 January 2021).

New York Department of Corrections and Community Supervision. DOCCS COVID-19 Report. <https://doccs.ny.gov/doccs-covid-19-report>. (Accessed 15 January 2021).

NC DPS. Previous Actions Taken. <https://www.ncdps.gov/our-organization/adult-correction/prisons/prisons-info-covid-19#actions>. (Accessed 15 January 2021).

North Dakota Corrections and Rehabilitation. Frequently Asked Questions. <https://www.docr.nd.gov/sites/www/files/documents/friends_family/FAQ.pdf>. (Accessed 15 January 2021).

Ohio Department of Rehabilitation and Corrections. (2020). COVID-19 Current Month Inmate Testing. <https://coronavirus.ohio.gov/static/reports/DRCCOVID-19Information.pdf>. (Accessed 15 January 2021).

Oklahoma Department of Corrections. (2020). Pandemic Planning Guide. <https://oklahoma.gov/content/dam/ok/en/doc/documents/administration/odoc-pandemic-planning-guide.pdf>. (Accessed 15 January 2021).

Oregon Department of Corrections. COVID-19 Tracking. <https://www.oregon.gov/doc/covid19/Pages/covid19-tracking.aspx>. (Accessed 15 January 2021).

Oregon Department of Corrections. ODOC COVID-19 Infection Prevention, Testing, and De-Escalation Protocol. <https://www.oregon.gov/doc/covid19/Documents/tiered-protocol-institutions.pdf>. (Accessed 15 January 2021).

Pennsylvania Department of Corrections. COVID Demobilization Plan,  <https://www.cor.pa.gov/Documents/PA-DOC-COVID-Demobilization-Plan.pdf>. (Accessed 15 January 2021).

Pennsylvania Department of Corrections. Coronavirus. <https://www.cor.pa.gov/Pages/COVID-19.aspx#Demobilization>. (Accessed 15 January 2021).

Rhode Island Department of Corrections. (2020). COVID-19 Inmate Quarantine & Isolation Protocols. <http://www.doc.ri.gov/documents/covid-19/inmate-quarantine-and-isolation-protocols-12-9-2020.pdf>. (Accessed 15 January 2021).

South Carolina Department of Corrections. (2020). South Carolina Department of Corrections (SCDC) COVID-19 Action Plan. <http://www.doc.sc.gov/scdc_covid-19_action_plan_031620.pdf>. (Accessed 15 January 2021).

South Dakota Department of Corrections. COVID-19 Frequently Asked Questions. <https://doc.sd.gov/documents/CoronavirusInformation9172020.pdf>. (Accessed 15 January 2021).

Tennessee Department of Correction. Frequently Asked Questions Regarding COVID-19. <https://www.tn.gov/correction/frequently-asked-questions-regarding-covid-19.html>. (Accessed 15 January 2021).

Texas Department of Criminal Justice. Definitions and Methodology. <https://www.tdcj.texas.gov/covid-19/definitions.html>. (Accessed 15 January 2021).

Utah Department of Corrections. COVID-19: What to expect at the Utah Department of Corrections. <https://corrections.utah.gov/images/Inmate_handout_full_0407_1.pdf>. (Accessed 15 January 2021).

Vermont Department of Corrections. COVID-19 GUIDELINES - Facility. .<https://doc.vermont.gov/sites/correct/files/documents/COVID-19%20Facility%20Protocol%20for%208-24-20%20to%20post.pdf>. (Accessed 15 January 2021).

Washington State Department of Corrections. WA State DOC COVID-19 Screening, Testing, and Infection Control Guideline Version 23. <https://www.doc.wa.gov/corrections/covid-19/docs/screening-testing-infection-control-guideline.pdf>. (Accessed 15 January 2021).

West Virginia Division of Corrections and Rehabilitation. COVID-19 Response Plan. March 20, 2020.<https://dhhr.wv.gov/COVID-19/Documents/COVID19_DCR_Response%20Plan-Policy_337-redacted_2020_03-20.pdf>. (Accessed 15 January 2021).

Wisconsin Department of Corrections. COVID-19 (Coronavirus): Persons in our Care Testing Dashboard. <https://doc.wi.gov/Pages/COVID19%28Coronavirus%29/COVID19TestingDashboard.aspx>. (Accessed 15 January 2021).

Gary Craig. COVID-19 outbreak at Wyoming prison could be harbinger of more to come. <https://www.democratandchronicle.com/story/news/2020/11/19/covid-19-outbreak-wyoming-prison-could-harbinger-more-come/6337883002/>.
